# Development and longevity of naturally acquired antibody and memory B cell responses against *Plasmodium vivax* infection

**DOI:** 10.1371/journal.pntd.0012600

**Published:** 2024-10-24

**Authors:** Pongsakorn Thawornpan, Piyawan Kochayoo, Zulfa Zahra Salsabila, Patchanee Chootong

**Affiliations:** Department of Clinical Microbiology and Applied Technology, Faculty of Medical Technology, Mahidol University, Bangkok, Thailand; Universidade do Estado do Rio do Janeiro, BRAZIL

## Abstract

*Plasmodium vivax* malaria causes significant public health problems in endemic regions. Considering the rapid spread of drug-resistant parasite strains and the development of hypnozoites in the liver with potential for relapse, development of a safe and effective vaccine for preventing, controlling, and eliminating the infection is critical. Immunity to malaria is mediated by antibodies that inhibit sporozoite or merozoite invasion into host cells and protect against clinical disease. Epidemiologic data from malaria endemic regions show the presence of naturally acquired antibodies to *P*. *vivax* antigens during and following infection. But data on the persistence of these antibodies, development of *P*. *vivax*-specific memory B cells (MBCs), and their relation to reduction of malaria severity and risk is limited. This review provides an overview of the acquisition and persistence of naturally acquired humoral immunity to *P*. *vivax* infection. Also, we summarize and discuss current progress in assessment of immune responses to candidate vaccine antigens in *P*. *vivax* patients from different transmission settings. Longitudinal studies of MBC and antibody responses to these antigens will open new avenues for developing vaccines against malaria infection and its transmission.

## Author summary

Despite the *Plasmodium vivax* parasite causing a large proportion of the global malaria burden, it has been neglected by much of the research world. Vaccines that can effectively induce strong and long-lasting antibody and memory B cell (MBC) responses are necessary for disease control and elimination, as they are crucial for blocking subsequent infection. The development and longevity of antibodies and MBC responses against different stages of *P*. *vivax* have been studied in various transmission settings. Some candidate antigens have been investigated in clinical trials with promising results. Previous reports have provided evidence of antibody acquisition to representative candidate *P*. *vivax* antigens from pre-erythrocytic, blood and transmission stages. However, evidence of durable antibody responses and development of *P*. *vivax*-specific MBCs is still lacking. Recently, expansion of atypical MBCs along with up-regulation of inhibitory receptors and reduced BCR signaling has been demonstrated and associated with short-lived antibody responses, culminating in impaired humoral immunity. Collectively, the capacity of *P*. *vivax* antigens to induce naturally acquired humoral immunity has been addressed. The results allow optimization of vaccine design to enhance immune responses to the parasite and protect against disease.

## Introduction

Malaria remains one of the most widespread and mortality-causing infectious diseases worldwide. *Plasmodium vivax* is the second most-prevalent cause of recurring malaria and infects millions of people each year, particularly in tropical and subtropical regions [1]. Being previously recognized as a causative agent of benign tertian malaria, recent data document that *P*. *vivax* can imitate the clinical severity and mortality of *P*. *falciparum* [2]. Moreover, additional serious issues associated with *P*. *vivax* infection are drug resistance [3] and clinical relapses [4]. Thus, development of an effective anti-*P*. *vivax* vaccine is considered an essential part of the overall strategy to reduce disease incidence, mortality, and morbidity.

The effort to develop malaria vaccines has been steadily ongoing. Yet, only the pre-erythrocytic vaccine RTS, S/ASO1 for *P*. *falciparum* has been licensed for human use [5]. There is no *P*. *vivax* vaccine available. Currently, only 3 *P*. *vivax* antigens [circumsporozoite protein (PvCSP), sexual-stage ookinete surface protein (Pvs25), and *P*. *vivax* Duffy-binding protein (PvDBP)] have reached clinical phases of vaccine development (Phases Ia and IIa/b) [6–8]. Since it remains unclear whether any of these candidate antigens confers protection, further knowledge of *P*. *vivax* antigenic diversity and pathways of immune evasion should augment vaccine development. In this review, we provide current and updated information from cross-sectional surveys and cohort studies in malaria endemic regions which demonstrate the responses of antibodies and memory B cells (MBCs) to *P*. *vivax* infection. Several studies have demonstrated the immunogenicity of *P*. *vivax* antigens (from pre-erythrocytic, asexual, and sexual stages) in eliciting humoral immune responses during *P*. *vivax* infection. However, the antigenic variation of these candidate antigens, leading to short-lived and strain-specific immunity, is a major obstacle to vaccine development. We also provide an update on the expansion and function of atypical MBCs which may impair long-lived antibody responses or produce antibodies upon receiving helper T cell signals. Such knowledge is essential for optimizing development of vaccines against *P*. *vivax*.

## Current status of *P*. *vivax* vaccines

Despite a number of tested *P*. *vivax* antigens from different parasite stages, very few vaccines have entered into clinical trials. Development of *P*. *vivax* vaccines has been hindered by the lack of a continuous culture system for the blood stage and limited availability of both ideal animal models to study parasite biology and access to fresh parasites from infected patients. Also, the *P*. *vivax* parasite has greater genetic diversity than does *P*. *falciparum* [9]. Together, these limitations represent a major hurdle for *P*. *vivax* vaccine development and the testing of vaccine efficacy. Both *P*. *falciparum* and *P*. *vivax* vaccines have been developed by focusing separately on the 3 stages of the parasite’s life cycle: (i) pre-erythrocytic stage (PE) vaccines which aim to halt infection by sporozoites recently introduced by a mosquito either before reaching the liver or before exiting this organ to invade red blood cells (RBCs); (ii) blood stage vaccines which target the asexual stage of the parasite by preventing entry into or reproduction in RBCs; and (iii) transmission-blocking vaccines (TBV) which aim to prevent the development of sexual forms within the mosquito.

The circumsporozoite surface protein (CSP) is the leading vaccine candidate for PE vaccine development because this protein is on the surface of mature sporozoites and fulfills a vital role for the parasite by supporting gliding motility and invasion of hepatocyte [10]. Genetic analyses revealed that PvCSP has 3 allelic variants (VK210, VK247, and *P*. *vivax*-like) [11]. The VMP001/AS01B vaccine, which consists of the N- and C- terminal regions of the PvCSP and a short repeat region present in both the VK210 and VK247 genotypes, has been evaluated in a Phase I/IIa trial and was able to induce antibody and cell-mediated immune responses [12]. In addition, 3 long synthetic peptides (LSPs) homologous to the N, central repeat, and C regions of PvCSP were evaluated in preclinical studies and showed high immunogenicity in mice and *Aotus* monkeys [13]. Then, these LSPs were formulated in Montanide ISA 720 and assessed in Phase Ia, IIa, and IIb clinical trials [14]. The vaccine proved to be safe, well-tolerated, and induced both cellular and humoral immune responses. However, the protective efficacy needs further study in larger volunteers as the protection showed 36% to 54.5% in malaria-naive volunteers (Phase IIa), while reduction of parasitemia in semi-immune volunteers (Phase IIb) did not show markedly difference from control group [14]. Recently, viral vector-based multistage vaccine composed of PvCSP-Pvs25 fusion protein showed long-term protection for more than 8 months against PvCSP-VK210 transgenic *P*. *berghei* sporozoites [15].

To date, 2 blood-stage vaccines have entered clinical trials. Both contain *P*. *vivax* Duffy Binding Protein II (PvDBPII), designated as ChAd63-PvDBPII/MVA-PvDBPII/Matrix-M and PvDBPII/GLA-SE [16]. The ChAd63-PvDBPII/MVA-PvDBPII/Matrix-M vaccine is prepared from recombinant viral vaccines using chimpanzee adenovirus 63 (ChAd63) and modified vaccinia virus Ankara (MVA) vectors. This vaccine induced antibody and T cell responses in Phase I/IIa clinical trial [16]. The vaccine-induced antibodies inhibited the binding of PvDBP region II to its receptor DARC. For the PvDBPII/GLA-SE vaccine, the results of a Phase I randomized trial to evaluate the safety and immunogenicity showed that the vaccine produced strain-transcending antibody responses that blocked receptor binding of diverse variant PvDBP alleles [17]. Thus, further clinical trials of the PvDBPII/GLA-SE vaccine to evaluate the efficacy against *P*. *vivax* in experimentally induced blood stage infection model or in natural infection will be useful [17]. As for transmission-stage vaccines, only 1 candidate, *P*. *vivax* surface protein (Pvs25), has been tested in a clinical trial (Phase I) [18]. This recombinant protein is produced from yeast cell expression system and combined with ISA-51 adjuvant. Anti-Pvs25 antibody levels peaked after the third vaccination. These vaccine-induced antibodies are functionally active, as evidenced by significant transmission blocking activity in a membrane feeding assay [8]. Moreover, anti-Pvs25 antibody concentration is correlated with the degree of inhibition.

Although *P*. *vivax* vaccine candidates have entered clinical trials, a satisfactory level of protection has not yet been achieved. It is undoubtedly necessary to look into details of the sero-epidemiological data related to different *P*. *vivax* antigens to inform down-selection of the most promising candidate antigens and to improve both the formulation and delivery systems to obtain greater immunogenicity. Furthermore, serological assays should be employed to survey the generation of naturally acquired antibody responses against *P*. *vivax* infection. Such information could provide a deeper understanding of the magnitude and persistence of humoral immunity to *P*. *vivax*.

## Naturally acquired antibody responses to *P*. *vivax* infection

Naturally acquired immunity developed after malaria infection is essential for protection against subsequent infections. Several studies support the current understanding of the development, durability, and protectivity of antibody responses (mostly IgG) against *P*. *vivax* infection generated by various antigens in the 3 life cycle stages: pre-erythrocytic stage, blood stage, and sexual stage. To date, it is unclear which malarial antigens are highly immunogenic and are most strongly correlated with protection against infection. In this section, we summarize current findings regarding development, maintenance, and protectivity of naturally acquired humoral immune responses to stage-specific vivax malaria antigens. The targets of naturally acquired antibodies reflect the presence of B cell epitopes which have and will guide vaccine design.

### Antibody responses against PE-stage antigens

The goal of PE-stage vaccine development is to block the early stage of *Plasmodium* sporozoite infection before completion of liver-stage development and breakthrough to the blood stage. However, little is known regarding the immune responses to antigens involved in hepatocyte invasion by sporozoites following natural infection. At present, serological responses against 3 PE antigens [circumsporozoite surface protein [19], thrombospondin-related anonymous protein (TRAP) [20], and cell-traversal protein for ookinetes and sporozoites (CelTOS)] in various transmission settings have been documented [21]. Analyses of naturally acquired antibodies to these PE antigens provide a basis for selection of potential candidate antigens to be included in *P*. *vivax* vaccine formulation [22].

PvCSP is the major surface protein of sporozoites that is directly exposed to host antibodies as these sporozoites migrate to the liver during the early phase of infection. Among 3 variants, PvCSP-VK210 is the major target of humoral immune responses. Serological studies in endemic settings have detailed the prevalence and magnitude of naturally acquired anti-PvCSP antibodies [19,23]. High titers of IgG responses against PvCSP-VK247 and PvCSP-VK210 strains were detected in individuals infected with *P*. *vivax* [11,23]. One study reported that anti-PvCSP antibody responses were positively correlated with parasitemia, but not age [24]. Cytophilic antibodies (IgG1 and IgG3), which appear to play a crucial role in forming a protective immune response are predominantly to this PE antigen [25]. Some studies pointed out that anti-PvCSP antibody response could be used as a tool for estimating past transmission. Anti-PvCSP antibodies were associated with HLA class II alleles, including HLA-DRB1*01, HLA-DQB1*02, and HLA-DQB1*05. This emphasizes the association between immunogenetic variation and antibody response [25].

The microneme proteins (PvTRAP and PvCelTOS) involved in sporozoite motility and invasion are also proposed as vaccine candidates [20,26]. PvTRAP is immunogenic in natural infections since *P*. *vivax* subjects residing in Iran, Afghanistan, Pakistan, and Brazil are seropositive to this antigen [26]. Responses of PvTRAP-specific IgG1 and IgG3 were stronger than those of other IgG subclasses, and the IgG3 response was positively correlated with length of time since the last malaria episode [26]. These data suggest that cytophilic IgG subclasses are involved in protection. Similarly, the immunogenicity of PvCelTOS has been documented in *P*. *vivax*-infected individuals in endemic regions of Colombia [21] and in western regions of Thailand [27]. One study group reported that among individuals residing in malaria endemic areas of Thailand, anti-PvCelTOS antibodies was the most frequent antibody compared to other pre-erythrocytic stage antigens [23]. Using predictive analyses, the half-life of anti-PvCelTOS antibodies is approximately 500 days [23]. Strikingly, inhibitory activity of anti-PvCelTOS antibodies is higher than that of PvCSP, PvSPECT1, and PvSSP3 antibodies [19].

In addition, other sporozoite proteins have been shown to induce immune responses. These include surface sporozoite protein 3 (SSP3), sporozoite microneme protein essential for cell traversal (SPECT1), sporozoite surface protein essential for liver-stage development (SPELD), and merozoite apical erythrocyte-binding ligand (MAEBL). A recent study demonstrated the immunogenicity of SSP3, SPECT1, SPELD, and the M2 domain of MAEBL by documenting specific antibodies in *P*. *vivax*-exposed residents living in an endemic region in Thailand [23]. Plasma samples from these *P*. *vivax*-infected subjects had inhibitory activity to *P*. *vivax* sporozoite invasion and liver-stage development, indicating that these antigens have vaccine potential in protecting against hepatocyte infection [23]. Altogether, PE antigens are promising given that they elicit antibody responses following naturally acquired infections. Future studies to confirm these target epitopes of anti-PE inhibitory antibodies and identify new ones, in combination with T cell epitopes, will be useful for development of vaccines to prevent the liver-stage development of *P*. *vivax* sporozoites.

### Antibody responses against blood-stage antigens

During blood-stage infection, successful host infection depends on specific receptor–ligand interactions between host red blood cells and *Plasmodium* parasites. Infective merozoites employ various proteins for attachment to and invasion of host red blood cells. These protein antigens are located at different locations of the apical complex and might be recognized by the immune system, culminating in the induction of antigen-specific antibody responses. Several studies have explored the antigenicity of blood-stage antigens, including surface, micronemal, and rhoptry proteins. Additional knowledge could be useful for design of vaccines against merozoite invasion and induction of immunity.

#### Merozoite surface proteins

The merozoite surface protein (MSP) family includes multiple forms of GPI-anchored proteins that mediate parasite invasion into host red blood cells [28]. Several studies congruently demonstrated the potent immunogenicity of multiple *P*. *vivax* antigens in the MSP family, including PvMSP1-19, PvMSP1-paralog (PvMSP1p-19), PvMSP-3, PvMSP-8, and PvMSP-9 [28–31]. Studies in malaria-endemic regions of Southeast Asia and the Brazilian Amazon showed high antigenicity of 19-kDa C-terminal regions of PvMSP1 (PvMSP1-19) [31,32] and its paralog (PvMSP1p-19) [29,33]. The magnitude of anti-PvMSP1-19 antibody responses was positively related to age and parasite density [31]. Similarly, a high seroprevalence of anti-PvMSP1p-19 IgG antibodies was detected among infected Thai individuals and cytophilic antibodies (IgG1 and IgG3 subclasses) were predominant [29]. The PvMSP3 (consisting of PvMSP3α, PvMSP3β, and PvMSP3γ) were immunogenic upon natural infection [34,35]. Anti-PvMSP-3α antibodies were associated with reduction of the burden of *P*. *vivax* malaria and protection against clinical disease [36]. Anti-PvMSP-9 antibodies are acquired in individuals residing in malaria endemic areas of Thailand; seropositivity was higher against the N-terminal than C-terminal domain, and the strength of response correlated with time since last malaria episode [30]. Altogether, the findings from *P*. *vivax*-infected patients strongly support the candidacy of PvMSP family members as components of a blood-stage vaccine.

#### Micronemal proteins

Several micronemal proteins function in *P*. *vivax* invasion of human erythrocytes [37]. Duffy Binding Protein II (DBPII) is a leading candidate antigen for a vaccine. Other candidates, such as the Apical Membrane Antigen 1 (AMA-1), Reticulocyte Binding Protein (RBP), GPI-anchored micronemal antigen (GAMA), and Erythrocyte Binding Proteins (EBPs), are also promising candidates. These blood-stage candidates show their immunogenicity in natural infections and animal models, as well as in clinical studies [16,38,39]. Since the mechanisms of *P*. *vivax* invasion of RBCs are not clear, documenting immunogenicity of the novel microneme proteins is required before proposing them as vaccine candidates.

PvDBPII is known to mediate the invasion preference of *P*. *vivax* for the Duffy antigen receptor for chemokines (DARC) [40]. DBP region II was reported to contain B cell epitopes [41]. Naturally acquired antibodies to PvDBPII are prevalent in residents of areas where malaria was highly endemic, but individuals differ significantly in the quantitative and qualitative nature of these responses [42]. The inhibitory activity of anti-PvDBP antibodies was positively correlated with age, suggesting that there is a boosting effect due to repeated infection [43]. Since PvDBPII is highly polymorphic, it is necessary to understand the breadth of anti-PvDBPII antibodies against the multiple allelic variants [44]. A study using plasma of infected individuals showed the presence of inhibitory antibodies which were cross-reactive against DBPII variants in Thailand, suggesting the presence of immunodominant conserved epitopes [45,46]. Moreover, B cell and T-cell epitopes have been characterized and found to be located at the DARC binding site and DBP dimer interface [41,47].

The high potential of PvAMA-1 to induce antibody responses in natural infection has been documented. In the Brazilian Amazon, acutely infected subjects had antibodies which strongly reacted with a synthetic PvAMA-1 peptide (residues S290-K307 and residues 43–487) [48,49]. An analysis of antibody responses to 3 distinct domains (Domains I, II, and III) of PvAMA-1 reveals that the strongest was to Domain II [50]. Recently, RBP-specific antibodies have gained much interest as a way to block invasion of immature red blood cells [51]. A high seroprevalence of anti-PvRBP1 antibodies was demonstrated in individuals from regions with natural exposure to *P*. *vivax* infections [52]. Naturally acquired anti-PvRBP1a and -PvRBP2c antibodies inhibited merozoite invasion of reticulocytes, indicating that PvRBP is also a candidate for inclusion as part of a blood-stage vivax vaccine [53]. Other micronemal proteins were able to induce antibody responses; PvGAMA is one of the glycosylphosphatidylinositol-anchored proteins (GPI-APs), known to be essential for merozoites invasion in malaria [54,55]. Cheng and colleagues reported inhibitory activity of sera from *P*. *vivax* patients against PvGAMA-mediated erythrocyte binding [55]. In addition, the immunogenicity of EBPs has been reported based on induction of naturally acquired antibody responses. Screening with a panel containing 342 *P*. *vivax* antigens, it has been shown that PvEBPII ranked among the top 9 antigens as targets of serum antibodies from children living in Papua New Guinea [56]. To date, there is no data showing inhibitory activity of anti-PvEBPII antibodies against merozoite invasion.

#### Rhoptry proteins

The rhoptry is a specialized organelle producing various proteins required for the red blood cell invasion process and parasitophorous vacuole formation. Structurally, a rhoptry consists of 2 distinct compartments: rhoptry neck and bulb. It has been reported that these proteins are involved in merozoite invasion in both *P*. *falciparum* and *P*. *vivax*. Four rhoptry proteins have demonstrated ability to induce anti-*P*. *vivax* immunity: (i) rhoptry-associated membrane antigen (RAMA) is a glycophosphatidylinositol (GPI)-anchored protein playing a role in rhoptry biogenesis and merozoite invasion; (ii) high molecular weight complex rhoptry protein-2 (RhopH-2) is essential for cytoadherence; (iii) rhoptry-associated, leucine zipper-like protein 1 (RALP1) is a rhoptry neck, erythrocyte-binding protein that contains a leucine zipper-like domain for protein–protein interactions; and (iv) rhoptry neck protein 2 (RON2) forms a complex with Apical membrane antigen 1 (AMA1) to begin junction formation followed by merozoite invasion [39,57,58].

The immunogenicity of *P*. *vivax* rhoptry proteins was demonstrated in natural infections. High seropositivity of anti-PvRALP1-Ecto and -PvRhopH2 antibodies were documented in *P*. *vivax*-infected subjects [39]. A study in Thai patients revealed that antibodies to PvRAMA were detected during acute malaria, and IgG3 was the predominant IgG subclass [58]. Also, high seropositivity of anti-PvRhopH2 IgG antibody was detected in *P*. *vivax*-infected subjects [39]. These anti-PvRhopH2 antibodies were short-lived, whereas specific MBCs persisted for at least 18 months after infection [39]. Moreover, antibodies against recombinant PvRALP1-Ecto proteins found in sera of *P*. *vivax*-infected subjects against both forms of PvRALP1-Ecto, suggesting the immunogenicity of PvRALP1-Ecto after malaria infection [39].

### Antibody responses to sexual stage antigens

Antigens intrinsically involved in sexual stages of the parasite in the gut of the mosquito (e.g., the gamete, zygote, and ookinete) are potential candidates for a transmission-blocking vaccine [59]. Sero-epidemiological studies help clarify humoral immune responses to sexual stage antigens (Pvs25, Pvs48/45, Pvs230, Pvs28) [59,60]. Immunological responses against Pvs25 and Pvs28 were mostly assessed by using sera of immunized mice, not infected humans [59]. One study revealed strong inhibitory activity of anti-Pvs25-positive sera, reducing the development of oocysts in mosquitoes [61]. Pvs230 appears highly conserved in nature, thus decreasing the possibility of potentially problematic strain-specific immunity. Antibody response against Pvs230 was acquired during *P*. *vivax* infection and the antibody titers tended to increase with age [62]. But compared to other sexual-stage antigens (such as Pvs25 and Pvs28), the protective activity of anti-Pvs230 antibodies is not yet well understood [62].

## Persistence of *P*. *vivax*-specific antibody responses postinfection

**Fig 1:**
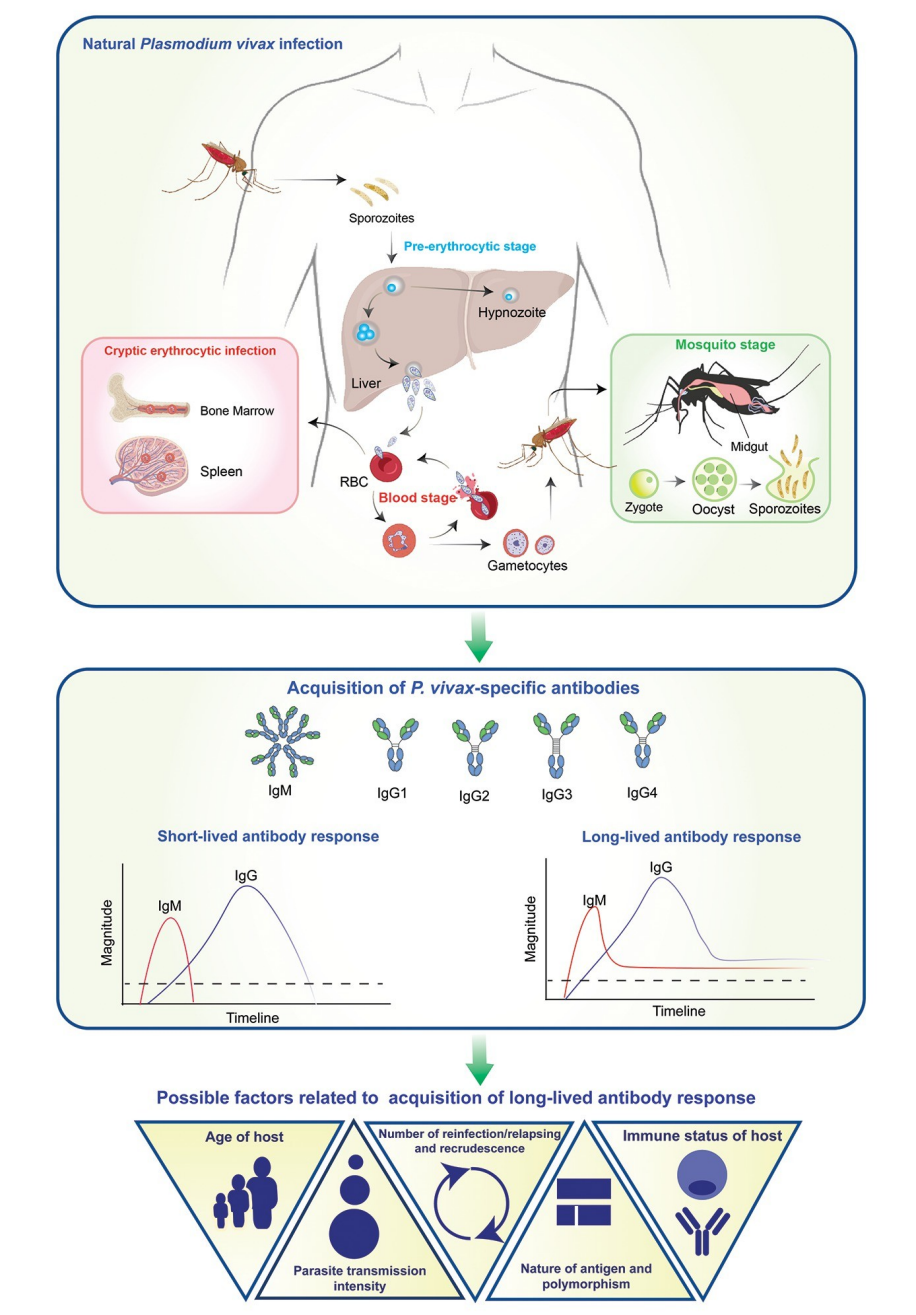
Development of antibody responses against *P*. *vivax* and the factors that might be involved in acquisition of long-lived antibody responses. **(Top)** *P*. *vivax* malaria can implicate 3 different stages of infection including pre-erythrocytic stage, blood stage, and mosquito stage. During the pre-erythrocytic stage, sporozoites infect and develop inside hepatocytes before releasing as merozoite or remain dormant by forming hypnozoite. In the blood-stage, merozoites invade and mature in red blood cells and might contribute to cryptic erythrocytic infection in spleen and bone marrow, thereby becoming sheltered from immune recognition and antimalarial drugs. In mosquito stage, male and female gametocytes can be taken up by a feeding *Anopheline* mosquito and form zygote in midgut. **(Middle)** In response to *P*. *vivax* infection, IgM antibodies are acquired at the initial phase of antibody response, being replaced by IgG antibodies in following weeks of the immunological memory phase. Antibody response could vary in different individuals, rendering short-lived or long-lived responses. Among 4 subclasses (IgG1-4), 2 predominant subclasses were IgG1 and IgG3. **(Bottom)** Possible factors related to the acquisition of long-lived antibody responses were proposed including host age, immune status, intensity of transmission, nature of encountering antigens (i.e., polymorphism and location of antigens), parasite recrudescence as well as number of reinfection/exposure.

The duration of acquired immune responses to malaria has been thought to be short [63,64]. However, long-lived *Plasmodium*-specific antibody responses in individuals with multiple infections have been detected [65]. Previous evidence suggested that the nature of an antigen [23], age of a host [66], immunogenetic status of the infected individual [67], and the number of re-infections/relapses [67] may affect the generation and persistence of anti-malarial antibodies (**Fig 1**). Greater understanding of the kinetics and duration of antibody responses following infection is required to improve vaccine efficiency and the design of reliable serological tools for surveillance of transmission.

To better determine the long-term immune responses in *P*. *vivax* malaria, sero-epidemiological studies have been conducted to assess the kinetics and duration of anti-*P*. *vivax* antibodies after recovery from infection. *P*. *vivax* antigens were reported to induce long-lived antibody responses following natural infection are summarized in **Table 1.** One study demonstrated that multiple *P*. *vivax* antigens (27 of 52 tested antigens) were highly immunogenic based on antibody responses at 1-week post-clinical *P*. *vivax* infection [68] and the maintenance of detectable IgG levels 9 months postinfection [68]. Changrob and colleagues showed that anti-PvDBPII antibodies have binding and neutralizing activities up to 9 months after *P*. *vivax* infection [69]. IgG antibodies against engineered synthetic DBPII (DEKnull-2) were boosted by recurrent blood-stage infections following treatment. In most cases of recurrent *P*. *vivax* infections, DEKnull-2 IgG antibodies were maintained for at least 12 months [70]. IgG responses to the synthetic peptides PvAMA-1 (S290-K307) and PvMSP-9 (E795-A808) were detected 6 months after infection [49]. Studies of other blood-stage antigens showed the presence of antibody responses against PvMSP1-19, PvMSP1P-19, PvMSP8, PvRBP1a, or rhoptry proteins at least 1-year postinfection [29,39,58,70–72]. A cohort study in Brazilian individuals detected antibody responses to 8 recombinant proteins showed that antibodies specific to PVX_081550 had the longest half-life, (100 days), followed by PvRBP2b (91 days) and Pv12 (82 days) [73]. In addition, a serological survey of antibodies specific to C-terminal region of PvMSP1 found that they persisted more than 5 months after infection [74].

**Table 1:** *P*. *vivax* antigens involved in induction of the longevity of antibody responses following natural infection.

No	Antigens	History of malaria episode (≥1 year)	Age (years)	Study area	Acute phase	Recovery phase	Ab responses			Persistence		Reference
							IgM	IgG	IgG subclasses	IgM	IgG	
**Pre-erythrocytic stage**												
1	PvCSP-210; PvCSP-247	0–1	32 (12–88)^a^	Brazil (low transmission)	-	> 12 months	-	✓	✓	-	≥12 months	[25]
2	PvCSP-VK210; PvSSP3; PvM2-MAEBL; PvCelTOS; PvSPECT1	0	29.5 (20–42)^a^	Thailand (low transmission)	✓	3, 9, and 12 months	-	✓	✓	-	≥12 months	[23]
3	PvTRAP; PvCyRPA; PvCelTOS	0-≥1	34.5 (7–64)^a^	Brazil (low transmission)	✓	6 months	✓	✓	✓	≥6 months	≥6 months	[79]
**Blood stage**												
1	Pv antigens (52 antigens)	0	29 (7–71)^a^	Thailand (low transmission)	✓	3, 6, and 9 months	✓	✓	✓	≥9 months	≥9 months	[68]
2	PvDBPII	0	37.4 ± 12^b^	Thailand (low transmission)	✓	3, 9, and 12 months	-	✓	-	-	≥12 months	[69]
3	PvDBPII DEKnull-2		27 (20–42)^a^	Brazil (low transmission)	✓	3, 6, and 12 months	✓	✓	-	-	≥12 months	[70]
4	PvMSP1-19		27 (20–42)^a^	Brazil (low transmission)	✓	3, 6, and 12 months	✓	✓	-	≥3 months	≥12 months	[70]
5	PvMSP1p-19	0	18–63^c^	Thailand (low transmission)	✓	3, 9, and 12 months	-	✓	-	-	≥9 months	[29]
6	PvMSP8	0	37.4 ± 12.0^b^	Thailand (low transmission)	✓	3, 9, 12, 36, and 48 months	-	✓	-	-	≥12 months	[71]
7	PvRBP1a	0	32.4 ± 14.4^b^	Thailand (low transmission)	-	3, 9, and 12 months	-	✓	-	-	≥12 months	[72]
8	PvRBP2b, PVX_081550, and Pv12	0	32.0 (12–64)^a^	Brazil (low transmission)	✓	1, 2, and 6 months	-	✓	-	-	≥6 months	[73]
9	PvRALP1-Ecto; PvRhopH2	1	40.1 ± 11.4^b^	Thailand (low transmission)	-	3, 9, and 12 months	-	✓	✓	-	3, 9, and 12 months	[39]
10	PvAMA1(S290-K307); PvMSP-9 (E795-A808)	0	29 (20–36)^a^	Brazil (low transmission)	✓	2 and 6 months	-	✓	-	-	2 and 6 months	[49]

^a^ Age is represented as median (IQR).

^b^ Age is represented as mean.

^c^ Age is represented as min-max.

Since the ability to relapse from dormant liver-stage hypnozoites of *P*. *vivax* parasite, understanding the effect of relapse on boosting immune responses will be useful for vaccine development. To date, the method to classify recrudescence, relapse or re-infection in *P*. *vivax* malaria is still limited. The lack of knowledge to define the cause of recurrent infection leads to difficulty in determining treatment efficiency, relapse rate, disease epidemiology as well as vaccine development strategy. Concerning association of number of clinical malaria episodes (no distinguishable relapse or reinfection) and antibody responses, a study in Brazil showed that the previous malaria episodes were associated with an increased anti-PvMSP1-19 IgG positivity [75]. The higher anti-PvMSP1 and -PvDBPII IgG antibodies were associated with one or more malaria episodes in individuals living in Peruvian Amazon [76]. In addition, a study in Thai endemic areas showed that the level and avidity of anti-PvMSP9 antibodies were positively related to the increasing malaria episodes [30]. Based on previous data, there is still a gap in knowledge on how the reactivation of the hypnozoites is related to the magnitude and durability of naturally acquired immune responses. Future studies to understand relapse patterns and their association with different components of the immune response will be directly relevant to the design of *P*. *vivax* malaria control strategies.

In contrast to blood-stage antigens, little is known about the longevity of anti-PE antibody responses. The persistence of anti-PvCSP-VK210 and PvCSP-VK247 IgG antibodies was observed over a 1-year period in regions of Brazil and Thailand. The results indicate that MBCs or long-lived plasma cells were capable of secreting IgG into the blood circulation of recovered patients [23,25,27]. In addition, high frequencies and titers of PvCelTOS IgG antibodies were found in subjects with acute malaria as well as 1 year postinfection. This highlights IgG responses as being a surrogate of exposures past as well as recent [23]. Recently, Thawornpan and colleagues showed that anti-PvSSP3 antibody responses have the longest half-life among 5 tested PE antigens (PvCSP-VK210, PvSSP3, PvM2-MAEBL, PvCelTOS, and PvSPECT1) [23]. Cytophilic IgG1 and IgG3 antibodies were the predominant subclass, tending to remain detectable for at least 360 days after infection [23]. Strikingly, there is an older report that anti-C-terminal PvCSP antibodies developed in infected individuals could persist for more than 30 years [77]. These findings raise more questions pertaining to the potential of these PE antigens to be vaccine candidates and thus require additional data on their immunological responses in humans.

In addition, longevity of IgM antibodies was observed in natural *P*. *vivax* infections. The detection of IgM responses to 30 *P*. *vivax* antigens in asymptomatic children from Papua New Guinea revealed that most tested proteins had seropositive IgM levels at week 0 and the antibody levels were consistently maintained over the 36 weeks [78]. A study in recurrence subjects who experienced 1 or 2 recurrent *P*. *vivax* infection in Brazil showed durable anti-PvMSP1-19 IgM at least 3-month follow-up period [78], whereas boosting effect was not detected in anti-EBPII and engineering DEKnull II IgM antibodies [70]. In Thai symptomatic subjects, IgM responses against 15 *P*. *vivax* antigens were maintained for at least 6 months in the absence of boosting infection, and the magnitude of the response was relatively similar for all tested *P*. *vivax* proteins [68]. Also, the seropositivity of IgM response to peptides of PE antigens (CelTOS, TRAP, and cysteine-rich protective antigen (CyRPA)) was found in a few individuals during acute malaria and after 30 or 180 days of infection [79]. Together, durability of IgM responses in *P*. *vivax* patients was detected. However, it still lacks data in the context of the association with malaria protection or decreasing clinical malaria severity as well as the mechanisms of *P*. *vivax*-induced IgM MBC or plasma cell responses.

## Generation and persistence of MBC responses to natural *P*. *vivax* infections

MBCs serve as major contributors to antibody production. However, to date it is still unclear what factors affect the maintenance of long-lived antibodies. Several efforts have been made to systematically monitor the longevity of MBC responses in individuals residing in areas with varying intensities of transmission of *P*. *vivax*. There is evidence that the production and persistence of *P*. *vivax*-specific MBCs depend on the differing parasite intensities in the transmission areas [39,71,72]. The *P*. *vivax* antigens that are reported to induce MBC responses following infection are summarized in **Table 2**.

**Table 2:** *P*. *vivax* antigens reported to induce MBC responses following natural infection.

No	Antigens	History of malaria episode (≥1 year)	Persistence	Age (years)	Study area	References
1	PvMSP8	0–1	4 years	37.4 ± 12.0^b^	Thailand (low transmission)	[71]
2	PvMSP1p-19	0	9 months	18–63^c^	Thailand (low transmission)	[29]
3	PvDBPII	0	9 months—3 years	37.4 ± 12^b^	Thailand (low transmission)	[69]
4	PvAMA-1; PvMSP1-19	0	6 year	19–48^c^	Thailand (low transmission)	[65]
5	PvRALP1-Ecto; PvRhopH2	1	18 months	40.1 ± 11.4^b^	Thailand (low transmission)	[39]
6	PvRBP1a	0	1–3 years	32.4 ± 14.4^b^	Thailand (low transmission)	[72]
7	PvAMA1	0	6 months	23.0–34.5^c^	Brazil (low transmission)	[86]
8	PvAMA1 (S290-K307); PvMSP-9 (E795-A808)	0	6 months	29 (20–36)^a^	Brazil (low transmission)	[49]

^a^ Age is represented as median (IQR).

^b^ Age is represented as mean.

^c^ Age is represented as min-max.

Accumulating evidence demonstrates the durability of blood-stage-specific MBCs. MBCs specific to PvMSP1-19 were found in *P*. *vivax* subjects whose responses were stably maintained for over 6 years without reinfection [65]. *P*. *vivax* patients residing in areas of low transmission had stable PvDBPII-specific MBC responses over 3 years without reinfection [69]. Moreover, MBCs specific to PvMSP1P-19, PvMSP8, Rhoptry protein (RALP1-Ecto and RhopH2), and PvRBP1a were maintained in Thai *P*. *vivax* patients for at least 9 months, 4 years, 18 months, and 3 years after infection, respectively [29,39,71,72]. A study performed in Brazil found that PvAMA1-specific MBCs were maintained in adult Amazonians for 6 months after parasite clearance [70]. Also, MBCs specific to the PvMSP-9 (E795-A808) protein were detectable without reinfection in all study groups through the 6-month follow-up period [49]. At present, data is scarce regarding the acquisition of MBC responses to PE antigens. The short-lived efficacy of the RTS, S vaccine against *P*. *falciparum* in children might indicate a low ability of the vaccine to induce immunological memory. Thus, the fundamental B cell responses to sporozoite proteins may be a major challenge for PE-based vaccine development both in *P*. *falciparum* and *P*. *vivax* malaria.

Altogether, *P*. *vivax* infection appears to induce MBC responses with long-term persistence postinfection. However, knowledge gaps remain in regard to the boosting effects needed to maintain *P*. *vivax*-specific MBCs: (i) whether relapse/reinfection promotes the durability of *P*. *vivax*-specific MBC responses, and if so (ii) what is the optimal time for boosting to enhance persistence of MBC responses. Future longitudinal studies to assess the immunogenicity of vaccine candidates should consider these factors and determine the immunodominant epitopes capable of triggering long-term *P*. *vivax*-specific MBCs. This knowledge will be critical for *P*. *vivax* vaccine development.

## Expansion of atypical MBCs in patients with *P*. *vivax* infection

**Fig 2:**
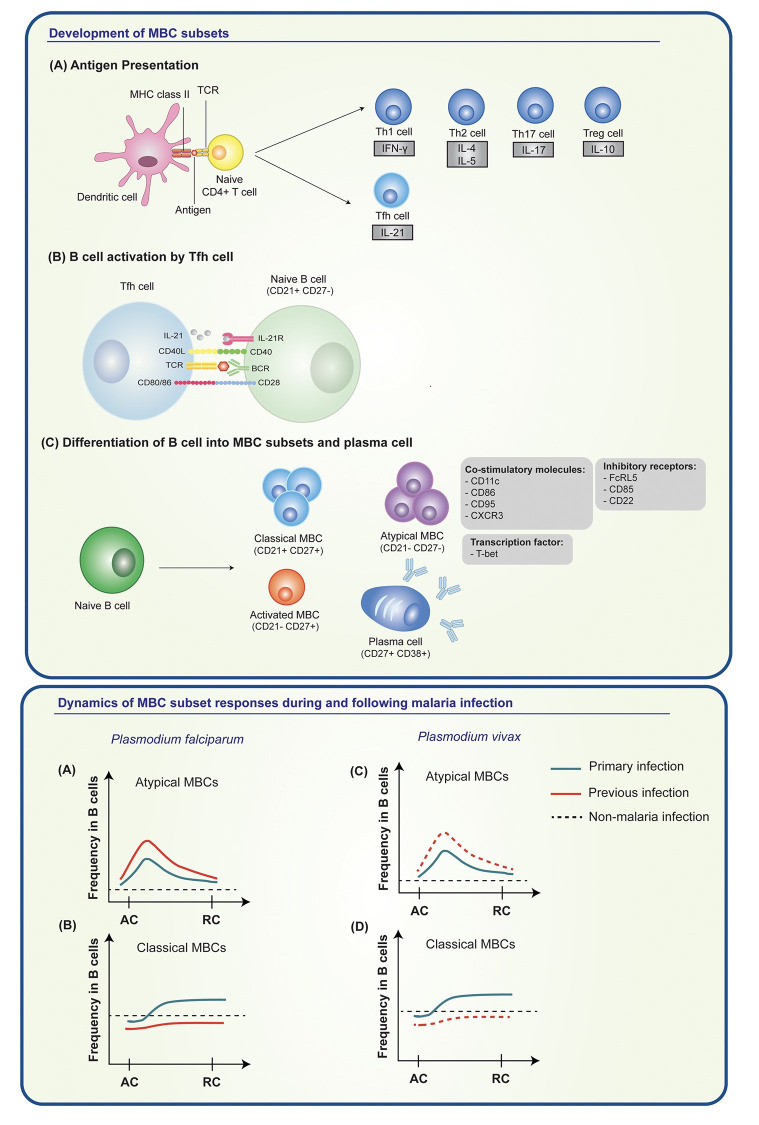
Development and dynamics of MBC subset responses in *P*. *vivax-*infected individuals during acute and recovery phase of infection. **(Top)** Following activation by encountering malaria antigen, dendritic cells induce the activation of naive T cells. The CD4^+^ T cells differentiate into effector T helper cells (Th1, 2 and 17), regulatory T and follicular T cells, which secrete characteristic cytokines including IFN-γ, IL-4/IL-5, IL-17, IL-10, and IL-21, respectively **(A)**. The interaction between follicular helper T (Tfh) and naive B cells through cytokine and co-stimulatory molecules drives B cell activation, leading to the generation of MBC subsets **(B)**. The phenotyping of MBCs reaveals 3 phenotypically distinct subsets, including CD21- CD27+ activated MBCs, CD21^-^CD27^-^ atypical MBCs and CD21^+^CD27^+^ classical MBCs **(C)**. Atypical MBCs were expanded during *P*. *vivax* infection. However, the functions of these cells in anti-malarial humoral immunity are still unclear. Several biomolecules related to atypical MBCs have been investigated, including co-stimulatory molecules (CD11c, CD86, CD95, CXCR3), inhibitory receptors (FcRL5, CD22, CD85), and T-bet transcription factor. **(Bottom)** The responses of atypical and classical MBCs during acute (AC) and recovery (RC) phase of *P*. *falciparum* and *P*. *vivax* infection are shown. In *P*. *falciparum* infection **(A, B)**, subjects with primary and previous infection showed an expansion of atypical MBCs during AC malaria, with higher frequency in previously infected subjects. These MBCs decreased but remained higher than frequencies of non-malaria subjects after recovery from infection. The frequencies of classical MBCs during AC infection were lower than baseline of non-malaria subjects. They increased in RC phase both in primary and previous infection. In cases of *P*. *vivax* infection **(C, D)**, individuals with acute primary infection exhibited an elevated frequency of atypical MBCs. Although this frequency decreased over time at RC phase, it remained higher than that observed in non-malaria infections. In contrast, primary infection showed lower number of classical MBCs during AC illness, compared to non-malaria infection. In RC phase, frequency of classical MBCs slowly increased. To date, the boosting response of both atypical and classical MBCs in previously *P*. *vivax*-infected individuals is still unclear. Solid green line represents primary infection, solid red line represents previous infection, dashed red line indicates unclear data in *P*. *vivax*, and dashed black line indicates non-malaria infection.

Malaria infection alters the distribution of circulating MBC populations. The expansion of atypical MBCs with CD21^-^CD27^-^ phenotypes has been reported in individuals from *P*. *falciparum* and *P*. *vivax* malaria-endemic areas [69,80,81]. The functional phenotyping of atypical MBCs in *P*. *falciparum* patients showed overexpression of co-stimulatory molecules (CD11c, CD86, CD95, and CXCR3) and inhibitory receptors (FcRL5, CD85, and CD22) [82,83], and reduction in phosphorylation of Syk and PI3K molecules in BCR signaling pathway, together indicating ineffective function of B cells in producing antibody [84]. A study in PNG showed higher proportions of IgD^-^ atypical MBCs in *P*. *vivax-*exposed women, compared to non-exposed individuals [85]. Atypical MBCs expressed more C-C Motif Chemokine Receptor (CCR3) and less IgG on the surface than did their resting counterparts, suggesting that they indeed have more with the activated phenotype [85]. In Brazil, patients with primary *P*. *vivax* infections had elevated frequencies of activated and atypical MBCs, while others who have experienced multiple malaria episodes display a lower proportion of atypical MBCs and higher frequency of classical MBCs [86,87]. Functional phenotyping of atypical MBCs shows high expression of T-bet, activation markers (CD11c, CD69), co-stimulatory molecules (CD86 and IL-21R), and FcRL5 molecules during malaria illness [80]. Regarding persistence of atypical MBCs, a cohort study in India found an expansion of atypical MBCs during acute malaria and the frequency of these cells to be decreased to baseline, 30 days after recovery [88]. In contrast, a study in Thai subjects recovered from vivax malaria maintained atypical MBCs for at least 3 years after infection [69]. Deep phenotype profiling of *P*. *vivax-*specific atypical MBCs is needed to determine whether this MBC subset plays a role in protection or suppression of humoral immunity. The development and dymamics of MBC subset responses following malaria infection are presented in **Fig 2**.

The role of atypical MBCs in malaria antibody production is unclear whether they activate or impair the generation of humoral immunity. Studies of *P*. *falciparum*-exposed individuals found negative effects of increased atypical MBCs on the development of long-lived antibody secreting cells, as well as on antibody production [89,90]. These cell populations decreased BCR signaling [82,84] and limited antibody secretion after in vitro stimulation [84]. However, a recent study showed that atypical MBCs selectively respond to membrane-bound antigens, but not to soluble antigens [91]. The comparison of atypical MBC responses to low-affinity versus high-affinity antigens showed minimal differences, indicating that atypical MBCs selectively reduced responses to low-affinity antigens [92]. A study recruiting *P*. *falciparum* patients in Malian revealed a function of atypical MBCs in plasma cell differentiation and immunoglobulin secretion after culture with polystimulator and follicular helper T (Tfh) cells. These data indicate a cooperative function between Tfh and atypical MBCs in the generation of antibodies [93]. Until now, only 1 study has demonstrated a function of atypical MBCs in *P*. *vivax* infection: atypical MBCs reduce Syk phosphorylation following BCR cross-linking. A combination of TLR-7/8 and T cell-derived cytokines (IL-21 and IFN-γ) is required to induce plasma cell differentiation and antibody secretion [80]. Although several research groups have attempted to demonstrate the function of this cell population in natural malaria exposure, the studies are complicated by the different genetic backgrounds of individual malaria patients, number of previous exposures and the different in vitro conditions (including triggers/signals) in stimulation cultures which could all impact function of these cells. Immunological signals and mechanisms that drive atypical MBC function remain to be identified.

## Conclusions

Developing an effective vaccine that provides long-term protection and prevents transmission is essential for the elimination of *P*. *vivax* malaria. Humoral immunity is the main mechanism of protection against malaria. Seroprevalence surveys in malaria endemic regions show that *P*. *vivax* infections significantly induce specific antibody responses. Across settings with different malaria endemicity, persistence of seropositive responses occurs at postinfection, indicating the potential of *P*. *vivax* antigens to induce MBC function. However, the association between longevity of anti-*P*. *vivax* humoral immunity and protection against malaria or reduced clinical severity is still very limited. There are large gaps in knowledge regarding the mechanisms and factors that can induce the development of durable antibody and MBC responses: (i) since only a few *P*. *vivax* subjects produce functional antibodies against parasite invasion, the factors that contribute to generation of inhibitory antibodies and MBC-secreted inhibitory antibodies such as number of re-infection/relapse, host genetic factors, and parasite densities needs to be demonstrated; (ii) it is unclear whether *P*. *vivax*-specific MBCs require specific malarial antigen re-stimulation or B cell receptor co-stimulation signaling to differentiate into antibody-secreting cells (ASCs); (iii) to avoid short-lived and strain-specific antibody and MBC responses, identifying conserved protective B cell epitopes sharing among *P*. *vivax* variant antigens is needed; (iv) in promoting the development of long-lived malaria-specific MBCs and plasma cells, understanding of the interactions of follicular helper T-B cells in generating anti-malarial antibodies is needed; (v) analysis of development of *P*. *vivax*-specific atypical MBCs (CD19^+^CD27^+^cells) whether this MBC subset is triggered from specific *P*. *vivax* antigen stimulation or condition during acute infection as well as demonstrate function of *P*. *vivax*-specific atypical MBCs in secreting protective antibodies is required. Overall, in-depth understanding of these gaps is fundamental for improving the effectiveness of *P*. *vivax* vaccines.

### Key Papers

- Changrob S, McHenry AM, Nyunt MH, Sattabongkot J, Han E-T, Adams JH, et al. Persistence of long-lived memory B cells specific to Duffy Binding Protein in individuals exposed to *Plasmodium vivax*. Scientific Reports. 2018;8(1):8347.
- Tashi T, Upadhye A, Kundu P, Wu C, Menant S, Soares RR, et al. Longitudinal IgG antibody responses to Plasmodium vivax blood-stage antigens during and after acute vivax malaria in individuals living in the Brazilian Amazon. PLoS Negl Trop Dis. 2022;16(11):e0010773. Epub 20221123.
- Kochayoo P, Thawornpan P, Wangriatisak K, Changrob S, Leepiyasakulchai C, Khowawisetsut L, et al. Interferon-γ signal drives differentiation of T-bet^hi^ atypical memory B cells into plasma cells following *Plasmodium vivax* infection. Scientific Reports. 2022;12(1):4842. 10.1038/s41598-022-08976-6.
- Wipasa J, Suphavilai C, Okell LC, Cook J, Corran PH, Thaikla K, et al. Long-lived antibody and B cell memory responses to the human malaria parasites, *Plasmodium falciparum* and *Plasmodium vivax*. PLOS Pathogens. 2010; 6(2):e1000770.
- Thawornpan P, Nicholas J, Malee C, Kochayoo P, Wangriatisak K, Tianpothong P, et al. Longitudinal analysis of antibody responses to *Plasmodium vivax* sporozoite antigens following natural infection. PLOS Neglected Tropical Diseases. 2024; 18(1):e0011907.

### Learning Points

- Development of *P*. *vivax* vaccines is ongoing and several vaccine candidates have entered clinical trials to investigate their safety and immunogenicity.
- Humoral immunity against *P*. *vivax* can be acquired following natural infection.
- *P*. *vivax* antibody persistence, MBC development, and their association with clinical protection against malaria remain incompletely understood.
